# Prognostic Accuracy of SpO_2_-based Respiratory Sequential Organ Failure Assessment for Predicting In-hospital Mortality

**DOI:** 10.5811/westjem.59417

**Published:** 2023-09-25

**Authors:** Daun Jeong, Gun Tak Lee, Jong Eun Park, Sung Yeon Hwang, Taerim Kim, Se Uk Lee, Hee Yoon, Won Chul Cha, Min Seob Sim, Ik Joon Jo, Tae Gun Shin

**Affiliations:** Sungkyunkwan University School of Medicine, Samsung Medical Center, Department of Emergency Medicine, Seoul, Korea

**Keywords:** Sequential Organ Failure Assessment scores, pulse oximetry, sepsis, respiratory failure

## Abstract

**Introduction:**

In this study we aimed to investigate the prognostic accuracy for predicting in-hospital mortality using respiratory Sequential Organ Failure Assessment (SOFA) scores by the conventional method of missing-value imputation with normal partial pressure of oxygen (PaO_2_)- and oxygen saturation (SpO_2_)-based estimation methods.

**Methods:**

This was a single-center, retrospective cohort study of patients with suspected infection in the emergency department. The primary outcome was in-hospital mortality. We compared the area under the receiver operating characteristics curve (AUROC) and calibration results of the conventional method (normal value imputation for missing PaO_2_) and six SpO_2_-based methods: using methods A, B, PaO_2_ is estimated by dividing SpO_2_ by a scale; with methods C and D, PaO_2_ was estimated by a mathematical model from a previous study; with methods E, F, respiratory SOFA scores was estimated by SpO_2_ thresholds and respiratory support use; with methods A, C, E are SpO_2_-based estimation for all PaO_2_ values, while methods B, D, F use such estimation only for missing PaO_2_ values.

**Results:**

Among the 15,119 patients included in the study, the in-hospital mortality rate was 4.9%. The missing PaO_2_was 56.0%. The calibration plots were similar among all methods. Each method yielded AUROCs that ranged from 0.735–0.772. The AUROC for the conventional method was 0.755 (95% confidence interval [CI] 0.736–0.773). The AUROC for method C (0.772; 95% CI 0.754–0.790) was higher than that of the conventional method, which was an SpO_2_-based estimation for all PaO_2_ values. The AUROC for total SOFA score from method E (0.815; 95% CI 0.800–0.831) was higher than that from the conventional method (0.806; 95% CI 0.790–0.822), in which respiratory SOFA was calculated by the predefined SpO_2_ cut-offs and oxygen support.

**Conclusion:**

In non-ICU settings, respiratory SOFA scores estimated by SpO_2_ might have acceptable prognostic accuracy for predicting in-hospital mortality. Our results suggest that SpO_2_-based respiratory SOFA score calculation might be an alternative for evaluating respiratory organ failure in the ED and clinical research settings.

Population Health Research Capsule What do we already know about this issue?

*Although PaO*_2_ *is a reference value in the Sequential Organ Failure Assessment (SOFA) score, it is often unavailable for non-ICU patients.*

What was the research question?

*Are respiratory SOFA scores estimated by SpO*_2_ *comparable to the conventional method for predicting in-hospital mortality?*

What was the major quantitative finding of the study?

*The AUROC of the SpO*_*2*_ *-based respiratory SOFA (0.772; 95% CI 0.754–0.790) was higher than that of the conventional method.*

How does this improve population health?

*Respiratory SOFA scores estimated by SpO*_2_ *might be an alternative way to evaluate respiratory organ failure in the emergency department and clinical research.*

## INTRODUCTION

Sepsis is a life-threatening organ dysfunction caused by a dysregulated host response to infection.^1^ A recent analysis estimated 11 million sepsis-related deaths worldwide, accounting for almost 20% of all global deaths.^2^ Sepsis continues to be a major burden to healthcare systems including emergency departments (ED), affecting one of every 120 ED visits.^3^ ^–^ ^6^ The most recent revision of the sepsis definition (Sepsis-3) stresses the defining feature of sepsis as a “dysregulated host response to infection” and emphasizes focus on quantification of organ dysfunction.^1^ ^,^ ^7^ The Sepsis-3 definition adopts the Sequential Organ Failure Assessment (SOFA) score as a measure of organ failure, and the clinical criteria of sepsis included acute change in SOFA score.^7^ ^,^ ^8^

While various scoring systems can be used for prognostication of suspected sepsis patients, the SOFA score is the most validated system and an essential component of a clinical sepsis definition.^9^ The SOFA score was initially designed to provide population-level insights into acute morbidity in intensive care unit (ICU) patients, but it has become integrated into many aspects of critical care in both ICU and non-ICU settings including the ED.^10^ The SOFA score is based on six organ categories, one for each of the respiratory, cardiovascular, hepatic, coagulation, renal, and neurological systems, each scored from 0 to 4, with an increasing score reflecting worsening organ dysfunction.^11^

The severity of respiratory dysfunction is measured with the SOFA score based on the ratio of partial pressure of oxygen (PaO_2_) to fraction of inspired oxygen (FiO_2_) (PF). The PF ratio provides information about pulmonary gas exchange adjusted for the quantity of oxygen delivered.^12^ Although PaO_2_ is a reference variable, invasive arterial blood gas (ABG) measurements are infrequently performed, and PF ratios are often unavailable for patients outside the ICU.^1^ Furthermore, PaO_2_ is often measured once rather than multiple times, which reduces clinical utility in non-ICU settings. In clinical studies, missing PaO_2_ values are usually considered normal. As a noninvasive alternative to PaO_2_, peripheral oxygen saturation (SpO_2_)-based estimation and the SpO_2_/FiO_2_ (SF) ratio have been proposed, but comparative data of estimation methods including simplified or mathematical models in non-ICU settings are limited and require further validation.^12^

In this study we aimed to investigate the prognostic accuracy for predicting in-hospital mortality of respiratory SOFA scores by the conventional method of missing value imputation with normal PaO_2_- and SpO_2_-based estimation methods.

## METHODS

### Study Design

This was a single-center, retrospective cohort study of patients with suspected infection who presented to the ED of a tertiary-care hospital located in a metropolitan city between December 2017–November 2019. This study was approved by the Institutional Review Board of Samsung Medical Center (No. SMC 2022-08-158-001). The requirement for informed consent was waived given the study’s retrospective nature and anonymized patient data. We followed the guidelines of the Strengthening the Reporting of Observational Studies in Epidemiology Statement (Appendix 1).

### Study Population and Definitions

We included patients ≥18 years old with suspected infection who presented to the ED. Suspected infection was defined as cases in which blood culture and antibiotic administration were conducted in the ED.^1^ ^,^ ^13^ We excluded patients who had limitations on invasive care (eg, patients who had terminal malignancy or who had previously signed a do-not-resuscitate [DNR] order), who presented with cardiac arrest, who had obvious non-infectious conditions such as trauma or bleeding, who were without SpO_2_ or FiO_2_, or had inadequate data due to our inability to access their electronic health record (EHR).

### Data Collection and Outcome Measurements

We collected retrospective cohort data by extraction from the hospital’s clinical data warehouse and review of EHR. Eligible cases were electronically identified by the aforementioned definition. Data extraction was carried out by two designated research coordinators trained on the definition of each variable by the investigator and who were blinded to the study hypothesis. To ensure high quality, one investigator reviewed the EHRs and verified the final data to resolve data conflicts. The following data were retrieved: demographic characteristics including age and gender; comorbidities; vital signs; laboratory data including platelet count, bilirubin, creatinine, lactate, and ABG analysis; vasopressor use; SOFA score; FiO_2_ and mechanical ventilation support; infection focus; and outcome-related data including in-hospital mortality and 28-day mortality. For collecting mortality data, we used visit history after discharge, mortality data provided by Statistics Korea, and telephone interviews. The primary endpoint was in-hospital mortality.

### Respiratory SOFA Score Assessment

Detailed equations for assessing respiratory SOFA score are shown in Table 1. As a conventional method, we calculated respiratory SOFA by PaO_2_ value and imputation as a normal value for missing PaO_2_. We used estimated PaO_2_ values from SpO_2_ based on two previously suggested methods (from Madan et al and Sauthier et al).^14^ ^,^ ^15^ We replaced all PaO_2_ (methods A and C) with estimated values regardless of the presence of measured PaO_2_, or we imputed missing PaO_2_ with estimated values (methods B and D). We also estimated respiratory SOFA scores by SpO_2_ and respiratory support use in all cases (method E) or in cases with missing PaO_2_ values (method F). We used a modified model from Valik et al because the original study did not incorporate use of respiratory support.^16^ All SOFA score components were calculated using maximum values during the 24 hours after ED arrival. Estimation of FiO_2_ in patients receiving supplementary oxygen is shown in Table S1.

**Table 1.** Respiratory SOFA assessment methods.

	Description	PaO_2_ and respiratory SOFA estimation	Reference
Conventional method	Missing PaO_2_ as normal	Normal value imputation	
Method A	SpO_2_-based estimation for all PaO_2_ values	1. For the first 10% reduction in SpO_2_ from 100% to 90%, decrease PaO_2_ by 4 mmHg for every percentage reduction in SpO_2_, with the resultant PaO_2_ decreasing from 100 to 60 mmHg	Madan et al.^14^
Method B	SpO_2_ based estimation for missing PaO_2_ values	2. For the next 10% reduction in SpO_2_ from 90% to 80%, decrease PaO_2_ by 1.5 mm Hg for each percentage reduction in SpO_2_, which will result in PaO_2_ decrease from 60 to 45 mm Hg.	
		3. For SpO_2_ levels below 80%, divide the value by 2.	
Method C	SpO_2_-based estimation for all PaO_2_ values	${\mathrm{PaO}}_{2}=(\frac{27.8^{2.8}}{\frac{1}{{\mathrm{SpO}}_{2}}-0.99}{)}^{\frac{1}{2.8}}$	Sauthier et al.^15^
Method D	SpO_2_-based estimation for missing PaO_2_ values		
Method E	Respiratory SOFA score estimation using SpO_2_ and respiratory support for all values	Respiratory SOFA calculation: Score 0: SpO_2_ >94% Score 1: 90 < SpO_2_ ≦94% Score 2: 85 < SpO_2_ ≦90% Score 3: SpO_2_ ≦85% *Add one point in each case for respiratory support such as oxygen or ventilator	Modified from the respiratory SOFA model of Valik et al.^16^
Method F	Respiratory SOFA score estimation using SpO_2_ and respiratory support for missing PaO_2_ values		

*SOFA*, Sequential Organ Failure Assessment; *PaO*_2_, partial pressure of oxygen in arterial blood; *SpO*_2_, peripheral oxygen saturation; *mm Hg*, millimeters of mercury.

### Statistical Analyses

Results are presented as median values with interquartile ranges (IQR) for continuous variables and numbers of patients with percentages for categorical data. Continuous and categorical variables were analyzed by the Kruskal-Wallis test and chi-square test, respectively. We compared prognostic performance of estimated respiratory SOFA score from each method with conventional respiratory SOFA score calculation for predicting in-hospital mortality. The estimated total SOFA scores from estimation methods for respiratory SOFA were compared to the total SOFA score by the conventional method. Discrimination was measured using the area under the receiver operating characteristic curve (AUROC). We also calculated the exact binominal 95% confidence interval (CI) for the AUROC. We measured the differences between conventional respiratory SOFA score AUROC and estimated respiratory SOFA score AUROC using the method proposed by DeLong et al.^17^ Calibration was assessed using calibration plots based on 100 bootstrap replicates. A *P*-value less than 0.05 was considered significant. We used R version 4.1.3 (R Foundation for Statistical Computing, Vienna, Austria; http://www.R-project.org/) for statistical analysis.

## RESULTS

### Study Population

**Figure 1.**
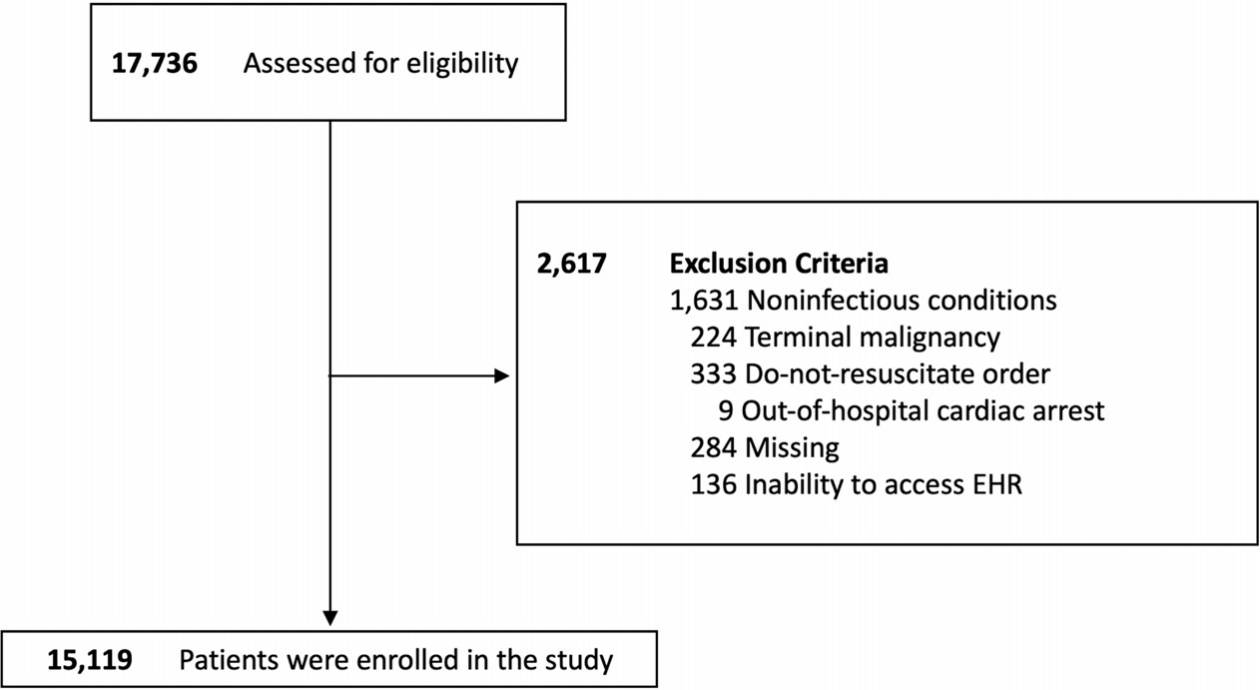
Study flowchart. *EHR*, electronic health record.

We assessed the eligibility of 17,736 adult patients who underwent blood culture and antibiotic administration in the ED from December 2017–November 2019. After excluding patients who had limitations on invasive care (eg, patients who had terminal malignancy or who had previously signed a DNR order), presented with cardiac arrest, had obvious non-infectious conditions such as trauma or bleeding, were missing data on SpO_2_ or FiO_2_, or had inadequate data due to inability to access the EHR, 15,119 patients were included in the analyses (Figure 1). As shown in Table 2, the overall median age was 63 years, and 8,248 of patients (54.6%) were male. Respiratory tract infection was the most common diagnosis, found in 4,523 patients (29.9%). The median PF ratio was 324.3 (IQR 255.2–388.1). The proportion of patients with missing PF ratio was 56.0%, and patients with data on PF ratio had higher in-hospital mortality (9.3% vs 1.4%; Table S2). The median SF ratio was 452.4 (IQR 443.0–461.9). Overall, the total conventional SOFA score was 2.0 (IQR 1.0, 4.0), and in-hospital mortality was 740 patients (4.9%).

**Table 2.** Baseline characteristics. The data are presented as median [IQR] for continuous variables or as number (%) for categorical variables.

Variables	Overall (N = 15,119)	In-hospital survival (n = 14,379)	In-hospital death (n = 740)	*P*-value
Age, years	63 [52, 73]	63 [52, 73]	66 [57, 75]	<0.01
Gender, female	6,871 (45.4)	6,597 (45.9)	274 (37.0)	<0.01
Comorbidities				
Hypertension	4,638 (30.7)	4,384 (30.5)	254 (34.3)	0.03
Diabetes	3,154 (20.9)	2,980 (20.7)	174 (23.5)	0.08
Cardiac disease	1,991 (13.2)	1876 (13.0)	115 (15.5)	0.06
Cerebrovascular disease	1,324 (8.8)	1,243 (8.6)	81 (10.9)	0.04
Chronic lung disease	1,370 (9.1)	1,277 (8.9)	93 (12.6)	<0.01
Hematologic malignancy	1,295 (8.6)	1,166 (8.1)	129 (17.4)	<0.01
Metastatic cancer	2,847 (18.8)	2,580 (17.9)	267 (36.1)	<0.01
Chronic renal disease	1,646 (10.9)	1,564 (10.9)	82 (11.1)	0.91
Chronic liver disease	1,316 (8.7)	1,233 (8.6)	83 (11.2)	0.02
Infection focus				
Respiratory tract	4,523 (29.9)	4,118 (28.6)	405 (54.7)	<0.01
Urinary tract	2,451 (16.2)	2,360 (16.4)	91 (12.3)	<0.01
Gastrointestinal	2,213 (14.6)	2,100 (14.6)	113 (15.3)	0.66
Hepatobiliary	2,633 (17.4)	2,562 (17.8)	71 (9.6)	<0.01
Bone or soft tissue	986 (6.5)	969 (6.7)	17 (2.3)	<0.01
Other focus	3,029 (20.0)	2,889 (20.1)	140 (18.9)	0.47
Unclear focus	662 (4.4)	630 (4.4)	32 (4.3)	1.00
Laboratory findings				
Platelets, 10^3^/L	197.00 [122.00, 273.00]	198.00 [127.00, 273.00]	130.00 [43.00, 249.00]	<0.01
Bilirubin, mg/dL	0.70 [0.40, 1.20]	0.70 [0.40, 1.20]	0.90 [0.50, 1.90]	<0.01
Creatinine, mg/dL	0.985 [0.766, 1.216]	0.84 [0.766, 1.14]	1.00 [0.71, 1.765]	<0.01
Lactate, mmol/L	1.56 [1.215, 2.325]	1.53 [1.14, 2.218]	2.42 [1.61, 4.34]	<0.01
Mean arterial blood pressure, mm Hg	75.00 [67.00, 83.00]	75.00 [68.00, 83.00]	67.50 [53.875, 78.00]	<0.01
Vasopressor use	1210 (8.0)	983 (6.8)	227 (30.7)	<0.01
PaO_2_, mm Hg	72.20 [61.40, 84.80]	72.90 [62.10, 85.30]	64.10 [54.70, 77.327]	<0.01
Missing PaO_2_	8462 (56.0)	8340 (58.0)	122 (16.5)	<0.01
PaO_2_/FiO_2_ ratio	324.329 [255.24, 388.10]	330.00 [264.876, 391.82]	248.657 [137.61, 326.43]	<0.01
SpO_2_	95.00 [93.00, 97.00]	95.00 [94.00, 97.00]	91.00 [85.00, 95.00]	<0.01
SpO_2_/FiO_2_ ratio	452.438 [442.986, 461.90]	452.438 [442.986, 461.90]	387.50 [219.876, 447.62]	<0.01
Mechanical ventilation	419 (2.8)	282 (2.0)	137 (18.5)	<0.01
Conventional respiratory SOFA (%)				<0.01
0	9,875 (65.3)	9,688 (67.4)	187 (25.3)	
1	2,581 (17.1)	2,433 (16.9)	148 (20.0)	
2	1,805 (11.9)	1,630 (11.3)	175 (23.6)	
3	580 (3.8)	450 (3.1)	130 (17.6)	
4	278 (1.8)	178 (1.2)	100 (13.5)	
Total conventional SOFA	2.00 [1.00, 4.00]	2.00 [1.00, 4.00]	6.00 [3.00, 10.00]	<0.01

*INR*, interquartile range; *SOFA*, Sequential Organ Failure Assessment; *L*, liter; *mg*, milligram; *dL*, deciliter; *PaO*_2_, partial pressure of oxygen in arterial blood; *SpO*_2_, peripheral oxygen saturation; *mm Hg*, millimeters of mercury; *FiO*_2_, fraction of inspired oxygen.

### Calibration of Respiratory SOFA Scores

**Figure 2.**
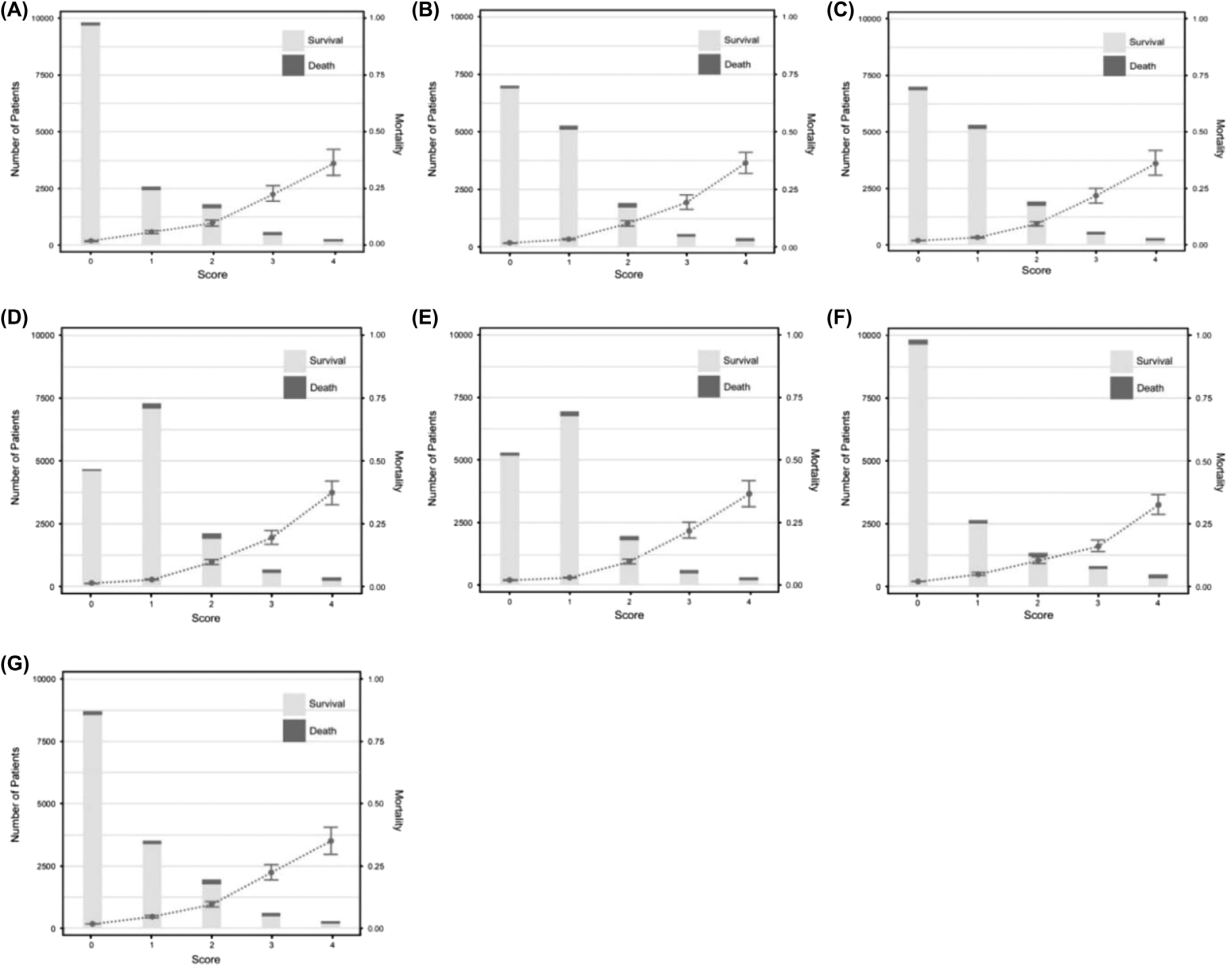
Distribution and in-hospital mortality according to respiratory SOFA scores by the conventional method and six estimation methods. Bar graphs represent number of patients, and points with error bars indicate in-hospital mortality with 95% confidence interval: (A) Conventional respiratory SOFA score. (B) Estimated respiratory SOFA score from method A. (C) Estimated respiratory SOFA score from method B. (D) Estimated respiratory SOFA score from method C. (E) Estimated respiratory SOFA score from method D. (F) Estimated respiratory SOFA score from method E. (G) Estimated respiratory SOFA score from method F. *SOFA*, Sequential Organ Failure Assessment.

Incidence and in-hospital mortality according to respiratory SOFA scores by the conventional method and the six estimation methods are shown in Figure 2. In-hospital mortality increased as estimated respiratory SOFA score increased in all methods. The calibration curve for in-hospital mortality showed similar calibration for all methods (Figure S1).

### Discrimination of Respiratory and Total SOFA Scores

The AUROCs of respiratory SOFA scores for predicting in-hospital mortality by the conventional method and by the six estimation methods are shown in Table 3 and Figure S2. The AUROC for method C (0.772; 95% CI 0.754–0.790) was significantly higher than that of the conventional method (0.755; 95% CI 0.736–0.773). The AUROCs of method B (0.739; 95% CI 0.719–0.759) and method D (0.735; 95% CI 0.715–0.755) were lower than that of the conventional method. The AUROCs of methods A (0.760; 95% CI 0.741–0.779), E (0.761; 95% CI 0.742–0.780), and F (0.758; 95% CI 0.739–0.777) were not significantly different from that of the conventional method.

**Table 3.** Area under the receiver operating characteristic curve for respiratory SOFA* scores for predicting in-hospital mortality by the conventional method and six estimation methods. *Conventional method respiratory SOFA score vs estimated respiratory SOFA score.

Respiratory SOFA score	AUROC	95% CI	*P*-value*
Conventional method	0.755	0.736–0.773	
Estimated methods			
Method A	0.760	0.741–0.779	0.47
Method B	0.739	0.719–0.759	0.02
Method C	0.772	0.754–0.790	0.02
Method D	0.735	0.715–0.755	0.01
Method E	0.761	0.742–0.780	0.38
Method F	0.758	0.739–0.777	0.42

*SOFA, Sequential Organ Failure Assessment; *AUROC*, area under the receiver operating characteristic curve; *CI*, confidence interval.

The AUROCs for total SOFA scores for predicting in-hospital mortality are shown in Table 4. The AUROC for total SOFA score from method E (0.815; 95% CI 0.800–0.831) was statistically higher than that for the conventional method (0.806; 95% CI 0.790–0.822). The AUROCs for methods B and D were lower than that of the conventional method. The AUROCs for methods A, C, and F were similar to that of the conventional method.

**Table 4.** Area under the receiver operating characteristic curve for total SOFA* scores for predicting in-hospital mortality by the conventional method and six estimation methods. *Conventional method total SOFA score vs. estimated methods total SOFA score.

Total SOFA score	AUROC	95% CI	*P*-value*
Conventional method	0.806	0.790–0.822	
Estimated methods			
Method A	0.807	0.791–0.823	0.77
Method B	0.796	0.779–0.814	<0.01
Method C	0.808	0.792–0.824	0.52
Method D	0.794	0.776–0.812	<0.01
Method E	0.815	0.800–0.831	<0.01
Method F	0.807	0.790–0.823	0.75

*SOFA, Sequential Organ Failure Assessment; *AUROC*, area under the receiver operating characteristic curve; *CI*, confidence interval.

## DISCUSSION

In this single-ED study of 15,119 patients with suspected infection, PaO_2_ values were commonly missing. Compared with a conventional missing value imputation with normal PaO_2_, SpO_2_-based estimation methods for missing PaO_2_ did not improve the prognostic accuracy for predicting in-hospital mortality. In contrast, respiratory SOFA scores estimated by SpO_2_, instead of measured and missing PaO_2_, yielded higher discrimination for respiratory SOFA assessment (method C using the equation from Sauthier et al) or total SOFA assessment (method E using a modified model from Valik et al). Our study showed that respiratory function assessment based on estimated respiratory SOFA scores from SpO_2_ is comparable to the conventional scoring system and could facilitate respiratory dysfunction assessment in the ED. Our study is important because we included patients with suspected infection in a non-ICU setting, where PaO_2_ measurement is limited but acute management of sepsis and septic shock usually take place.

The SOFA score is a validated tool for organ failure assessment and for defining clinical sepsis.^1^ ^,^ ^7^ The association of SOFA score with clinical outcomes has led many investigators to propose it as a potentially valid surrogate in clinical trials.^3^ ^,^ ^9^ However, accurate respiratory SOFA score evaluation requires an invasive ABG measurement, which is not routinely ordered in patients outside the ICU due to limited resources and substantial risk of failure or complications.^3^ Jakobsen et al and Gadrey et al addressed the issue that multiple imputations of large proportions of missing data lead to unreliable outcomes.^18^ ^,^ ^19^ SpO_2_ measured by pulse oximetry is a non-invasive, surrogate marker for tissue oxygenation that is routinely applied to most ED patients, and it can be monitored continuously.^20^ ^,^ ^21^ Previous studies introduced methods for imputing PaO_2_ from SpO_2_. Rice et al found that the SF ratio correlates with a simultaneously obtained PF ratio in acute respiratory distress syndrome.^22^ Sauthier et al developed and validated a method to filter SpO_2_ streams to estimate PaO_2_ using only continuous and noninvasive data.^15^ Valik et al showed that discrimination of mortality causes using SOFA score with respiratory function assessment based on SpO_2_ is comparable with that of conventional respiratory function assessment.^16^

All six estimated methods in our study replaced PaO_2_ regardless of the presence of measured PaO_2_ and yielded higher AUROCs for predicting in-hospital mortality. It is unclear why replacement of all PaO_2_ values with estimated SpO_2_ yielded better mortality-discriminant power than imputation of only missing PaO_2_ values. It may be because it is difficult to perform ABG sequentially in the ED. As it suggests, sequential increases in SOFA score are associated with organ dysfunction.^23^

Selection of the lowest SpO_2_ values from continuous monitoring might reflect deterioration in respiratory function better than does one-time PaO_2_ measurement. SpO_2_ measurement could identify more high-risk patients, including less severe patients, in the absence of PaO_2_ values (Table S2). An optimal strategy or equation to assess respiratory SOFA score can be selected considering the clinical settings, severity of patients, and number of PaO_2_ measurements. For example, we suggest that a simplified equation might be useful in resource-limited, urgent clinical settings like EDs. Among the six methods, Method E might be a good option for use in an ED. For clinical research, Method C would be preferred to show detailed data about estimated PaO_2_ and betted discrimination performance of respiratory SOFA score.

## LIMITATIONS

This study has several limitations. First, this was a single-center study conducted in the ED. Second, we were unable to assess pulse oximetry accuracy. There was the possibility that patient factors, such as skin pigmentation and peripheral circulation, affected SpO_2_ measurement. Third, there might have been a selection bias in acquiring ABG measurements. For generalizability, further studies including representative patients in non-ICU settings are needed to determine the proper relationship between PaO_2_ and SpO_2_.

## CONCLUSION

Our study shows that respiratory SOFA scores estimated by SpO_2_ might have acceptable or higher prognostic accuracy for predicting in-hospital mortality in ED patients with suspected infection who had not routinely undergone arterial blood gas analysis for PaO_2_ measurement. These findings suggest that SpO_2_-based respiratory SOFA score calculation might be an alternative way to evaluate respiratory organ failure in the ED and clinical research. Further studies for validation and modification of SpO_2_-based respiratory SOFA are needed.

## Supplementary Information




