# Supplementary material for: Prognostic Accuracy of SpO2-based Respiratory Sequential Organ Failure Assessment for Predicting In-hospital Mortality
Source: West J Emerg Med. 2023 Sep 25;24(6):1056–63. doi: 10.5811/westjem.59417 (PMC10754194; doi:10.5811/westjem.59417)
Supplement: Supplementary file 2 [file wjem-24-1056-s002.docx]

**Supplementary Table 1. Estimated FiO_2_ in patients receiving ventilatory support**

| **Estimated FiO_2_ in patients supported with low flow nasal cannula** | |
| --- | --- |
| Flow rate (L/min) | Estimated FiO_2_ |
| 1 | 0.24 |
| 2 | 0.27 |
| 3 | 0.3 |
| 4 | 0.33 |
| 5 | 0.36 |
| 6 | 0.39 |
| 7 | 0.42 |
| 8 | 0.45 |
| **Estimated FiO_2_ in patients supported with facemask** | |
| Flow rate (L/min) | Estimated FiO_2_ |
| 5 | 0.4 |
| 6-7 | 0.5 |
| 7-8 | 0.6 |
| **Estimated FiO_2_ in patients supported with facemask with reservoir bag** | |
| Flow rate (L/min) | Estimated FiO_2_ |
| 6 | 0.6 |
| 7 | 0.7 |
| 8 | 0.8 |
| 9 | 0.9 |
| 10+ | 0.95 |
